# cGMP and cardiac hypertrophy

**DOI:** 10.1186/2050-6511-16-S1-A12

**Published:** 2015-09-02

**Authors:** Ruwan K Perera, Hariharan Subramanian, Alexander Froese, Daniela Hübscher, Konrad R Götz, Viacheslav O Nikolaev

**Affiliations:** 1 Institute for Experimental Cardiovascular Research, University Medical Center Hamburg-Eppendorf (UKE), D-22529 Hamburg, Germany; 2 Heart Research Center Göttingen, University of Göttingen Medical Center (UMG), Göttingen, Germany

## Background

cGMP as a second messenger regulates cardiac contractility and might protect the heart from hypertrophy and failure by acting in distinct subcellular microdomains. However, direct visualization of cGMP in subcellular microdomains of adult cardiomyocytes has been challenging. Little is known also about changes in cardiomyocyte cGMP signalling at an early stage of the disease.

## Methods

We used a highly sensitive cytosolic and membrane-targeted Förster resonance energy transfer (FRET)-based biosensors for cGMP and cAMP for real time measurements in freshly isolated sensor-trasngenic adult ventricular cardiomyocytes. Combined with single cell contractility measurements, biochemical techniques and whole-heart recording, the effect of atrial natriuretic peptide (ANP) on cardiomyocyte cGMP/cAMP and contractility in healthy and hypertrophied hearts (after transverse aortic constriction).

## Results

Contractility measurements in hypertrophied hearts have unravelled ANP-induced augmentation of catecholamine stimulated increase in force and frequency of contraction which was present only in diseased hearts at the state of early compensated cardiac hypertrophy. Interestingly, this effect was not due to changes in cGMP content, membrane receptor densities or whole-cells phosphodiesterase (PDE) activity. Instead, physical redistribution of the cGMP-stimulated PDE2 and cGMP-inhibited PDE3 between distinct membrane domains led to a change of cAMP compartmentation towards an ANP/cGMP/PDE3-dependent increase of local cAMP levels in a microdomain regulating cardiac contractility [1]. FRET-based cGMP measurements revealed relatively small increase of cGMP upon ANP stimulation [2], stringent compartmentation of ANP/cGMP signals to T-tubular membranes and apparent absence of receptor desensitization in early cardiac hypertrophy [1].

## Conclusion

ANP/cGMP signalling can play distinct roles in cardiac disease, including a previously unrecognized contractility augmentation in early hypertrophy which might support heart function upon pressure overload. This can be achieved by PDE2/3 redistribution-dependent changes in cGMP/cAMP compartmentation.
